# Influence of center-based child care on development of two-year-olds in a Brazilian cohort

**DOI:** 10.11606/s1518-8787.2021055002958

**Published:** 2021-06-18

**Authors:** Otávio Amaral de Andrade Leão, Gregore Iven Mielke, Mariângela Freitas da Silveira, Marlos Rodrigues Domingues, Joseph Murray, Nelson Arns Neumann, Caroline Caus Dalabona, Andréa Dâmaso Bertoldi

**Affiliations:** I Univerisdade Federal de Pelotas Faculdade de Medicina Programa de Pós-Graduação em Epidemiologia PelotasRS Brasil Univerisdade Federal de Pelotas. Faculdade de Medicina. Programa de Pós-Graduação em Epidemiologia. Pelotas, RS, Brasil; II The University of Queensland School of Human Movement and Nutrition Sciences BrisbaneQueensland Australia The University of Queensland. School of Human Movement and Nutrition Sciences. Brisbane, Queensland, Australia; III Universidade Federal de Pelotas Escola Superior de Educação Física Programa de Pós-Graduação em Educação Física PelotasRS Brasil Universidade Federal de Pelotas. Escola Superior de Educação Física. Programa de Pós-Graduação em Educação Física. Pelotas, RS, Brasil; IV Human Development and Violence Research Centre PelotasRS Brasil Human Development and Violence Research Centre (DOVE). Pelotas, RS, Brasil; V Coordenação Nacional da Pastoral da Criança CuritibaPR Brasil Coordenação Nacional da Pastoral da Criança. Curitiba, PR, Brasil

**Keywords:** Child Care, organization & administration, Child Development, Health Development, Cohort Studies

## Abstract

**OBJECTIVE:**

To investigate the association between child care attendance since birth and development in two-years-old Brazilian children.

**METHODS:**

The study used longitudinal data from the 2015 Pelotas Birth Cohort. The childhood development (cognitive, fine and gross motor skills, and language) at two-years-old children was assessed using INTER-NDA (INTERGROWTH-21st Neurodevelopment Assessment). The child care attendance was measured at ages one and two years and categorized as: a) never attended child care; b) attended some child care (one or two years); and c) always attended child care (one and two years). Demographic, socioeconomic, health, and child stimulation variables were considered as confounders. Crude and adjusted analyses of child care attendance and development were carried out using linear regression.

**RESULTS:**

Out of the 3,870 infants included in the analyses, around 1/3 attended center-based child care. In crude analyses, attending center-based child care was associated with positive developmental outcomes, except in motor domains. In adjusted analyses, compared to those children that have never attended child care, children who did attend presented higher scores for cognitive development (always in child care: β: 2.44, 95%CI: 0.83–4.05; some child care: β: 1.35, 95%CI: 0.17–2.53).

**CONCLUSIONS:**

This study suggests that center-based child care may help improve child cognitive development in the Brazilian context. Furthermore, the association was higher for early and continued attendance. Considering the low prevalence of children in external care, it is recommended to improve child care opportunities in early childhood.

## INTRODUCTION

The first years of life are the most important period for growth and development of a human being, with a major impact on future health^1^. Child development is an interactive and maturational process, with the environment affecting multiple domains of development, including motor, language, cognitive, socio-emotional, and self-regulation skills^2^.

Recent evidence suggest that, in low and middle income countries, about 80 million children aged three and four years, experienced low cognitive and/or socio-emotional development in 2010^3^. A more recent study, including data from over 330,000 children from 63 low- and middle-income countries, found that a quarter of them were suspected to have developmental delay^4^. Thus, strategies to promote adequate development such as nurturing care, physical health, adequate early learning opportunities, and security and safety are critical in these countries^5^.

A large body of work has considered how child care environments can influence developmental outcomes. Early educational interventions show that preschool education can positively affect a child cognitive and social skills and school progress^6,7^, as well as behavioral, health, and schooling benefits^7^. The impact of early education and care programs – based on care centers – have shown short-term effects and small positive long-term effects on cognitive development^8,9^, social-emotional development, school progress, antisocial behavior, and even crime^9^. In Brazil, a study that evaluated the impacts of state public child care did not find associations between child care attendance and cognitive or executive function^10^.

Considering the fourth Goal of the Sustainable Development Goals, *“by 2030, ensure that all girls and boys have access to quality early childhood development, care and preprimary education so that they are ready for primary education”*^11^, and that the literature in low- and middle-income countries is still scarce – especially with a longitudinal focus – it is important to understand the role of center-based child care attendance for early child development (ECD). This study seeks to investigate the association between child care attendance from birth to two years and development at two years in a large population-based cohort study in Brazil.

## METHODS

### Study Design and Population

We analyzed data from the 2015 Pelotas (Brazil) Birth Cohort Study. Pelotas is a city in Southern Brazil, with around 340,000 inhabitants. All hospital-delivered children who were live-born in Pelotas from January 1^st^ and December 31st 2015, whose mother lived in the city urban area, were eligible for the study^12^. From the 4,333 eligible live births, 4,275 were assessed at birth (response rate 98.7%). All these children and their mothers were invited to participate in follow-up assessments at three, 12, and 24 months. Further information about the 2015 Pelotas Birth Cohort is available elsewhere^12^.

### Two-Year Follow-Up

In the two year follow-up, evaluations were carried out in a research clinic, with a 95.4% follow-up rate. Mothers answered a questionnaire updating previous information about the child’s and mother’s health, household characteristics, diseases, child activities, among other topics. Interviews and exams were conducted by female interviewers trained and retrained during the data collection phase. Informed consent form was obtained for participation in all phases of the study. The 2015 Pelotas birth cohort study was approved by the Research Ethics Committee of the School of Physical Education of Federal University of Pelotas (registration No. 26746414.5.0000.5313).

### Infant Development

Infant development was assessed when children were between 21 and 27 months old using the INTER-NDA (INTERGROWTH-21st Neurodevelopment Assessment)^13^. It consists of 53 items that are directly administered, concurrently observed and caregiver reported. INTER-NDA was designed to be free from cultural biases and it is based upon objective reporting of the child’s performance on cognition, expressive and receptive language, gross and fine motor skills, behavior, attention, and social-emotional reactivity. The selection of individual items were based upon expert’s agreement in suitability for age group, appropriateness for use in international populations, ability to be reliably administered and to be adapted across cultural contexts^13^

This instrument presents Interclass coefficient ranges between 0.75 and 0.83 (p < 0.001) and a moderate agreement with the Bayley Scales of Infant Development III (k = 0.72, p < 0.001), a well-established child development assessment, measuring cognition, language skills, motor skills, and adaptive behavior from one to 42 months^14^.

Assessment of children who did not present any serious physical or neural disability, precluding them from undergoing the INTER-NDA assessment, was conducted by trained interviewers, not experts in neurodevelopment, when children went to the clinic (excluding the phone interviews), or at the family’s home. Mean standardized scores were calculated for cognition, language, and fine and gross motor skills.

### Center-Based Child Care

Child care outside the home was measured using interview data from the one and two-years follow-up assessments. In the one year follow-up, mothers were asked about who took care of their children from birth to one year of age, and children receiving any center-based child care in that period were identified. At two years, mothers reported whether children were currently receiving any center-based child care. Those who responded “no” were also asked if the child had attended any center-based child care since the age of one year, although they were not currently attending.

For our analyses, center-based child care was then categorized into three mutually exclusive categories: a) never went to child care; b) attended some child care (either at one or two years only); c) always (both one and two years).

### Confounders

Covariates measured in the perinatal interview in this study were: child sex (female/male), maternal age (≤ 20, 21–30, and ≥ 31), family income (quintiles), maternal education (0–4, 5–8, 9–11, and 12+ years of schooling), pre-term (< 37 weeks), and low birth weight (< 2500g). Additionally, maternal depression was measured using the Edinburgh Postnatal Depression Scale (EPDS)^15^ – at the three-month follow-up – with a cut-off point of ≥ 13 points used to indicate the presence of at least moderate depression^16^.

In the two-year follow-up, mothers were asked about a series of indicators of child stimulation – if anyone read or told stories to their child (yes/no), if the child visited the house of other people in the past week (yes/no), if they went to a park in the last week (yes/no), and also if the child participated in the PIM (*Primeira Infância Melhor* - Better Early Childhood) program – a government home visitation initiative that seeks to enhance child development in vulnerable households with young children.

### Statistical Analysis

The analyses were conducted using Stata 16.0 in five steps: (a) descriptive analyses of children and their mothers were conducted for the entire cohort and compared with those with valid outcome data; (b) description of the participants, according to child care attendance; (c) description of the participants according to child development; (d) crude analysis of the relation between child care attendance and child development outcomes; (e) adjusted analysis of the relationship between child care attendance and child development outcomes.

For stage b, chi-squared tests were used. In steps c, d and e, linear regression models were used. For step e, adjusted analyses were conducted including, simultaneously, all covariates except child stimulation markers, in Model I. Model II also included variables related to child stimulation. Statistical significance was set at 5%, and 95% confidence intervals are provided.

## RESULTS

Of the 4,275 children enrolled in the original cohort, 3,870 had developmental data at age two years and were included in the analyses. Mothers of children included in the analyses were mostly 21–30 years old, had 9–11 years of education and about 10% presented depression symptoms. About 15% of the children were born preterm and 10% had a low birth weight. Regarding stimulation markers at age two years, about half of the children were read or told stories to, almost 3/5 went to parks, and more than 4/5 went to other people houses. About 10% of children were participating in the PIM program (Table 1).

**Table 1:** Descriptive characteristics of the study sample and Pelotas birth cohort sample.

Characteristic	Cohort characteristic (n = 4,275)	Inter-NDA sample (n = 3,870)
	n (%)	n (%)
Sex		
Male	2,164 (50.6)	1,965 (50.8)
Female	2,111 (49.4)	1,905 (49.2)
Mother’s age		
≤ 20	805 (18.8)	719 (18.5)
21–30	2,067 (48.4)	1,883 (48.7)
≥ 31	1,402 (32.8)	1,268 (32.8)
Income		
1 (low)	796 (19.8)	716 (19.6)
2	807 (20.1)	732 (20.1)
3	804 (20.0)	741 (20.3)
4	895 (22.3)	820 (22.5)
5 (high)	714 (17.8)	636 (17.5)
Maternal education (years)		
0–4	391 (9.2)	342 (8.9)
5–8	1,095 (25.6)	1,003 (25.9)
9–11	1,458 (34.1)	1,344 (34.7)
≥ 12	1,330 (31.1)	1,180 (30.5)
Pre term (< 37 weeks)		
Yes	663 (15.5)	570 (14.7)
No	3,612 (84.5)	3,300 (85.3)
Low birth weight (< 2,500g)		
No	3,830 (89.9)	3,497 (90.4)
Yes	428 (10.1)	371 (9.6)
Maternal depression (3m)		
No	3,642 (88.9)	3,395 (89.0)
Yes	453 (11.1)	420 (11.0)
Storytelling (2y)		
No	1,912 (47.7)	1,851 (47.9)
Yes	2,099 (52.3)	2,016 (52.1)
Child visits parks (2y)		
No	1,678 (41.8)	1,629 (42.1)
Yes	2,333 (58.2)	2,238 (57.9)
Child visits other houses (2y)		
No	638 (15.9)	592 (15.3)
Yes	3,373 (84.1)	3,275 (84.7)
Participation in PIM program (2y)		
No	3,637 (90.7)	3,500 (90.5)
Yes	374 (9.3)	367 (9.5)

Note: Highest number of missing for income (n = 3,645).

Table 2 shows that children who attended center-based child care were more likely to be boys, children with low birth weight, and children whose mothers were older, richer, more educated, and without elevated symptoms of depression. In addition, children who were stimulated through storytelling, taken to a park and those who did not participate in PIM were more likely to be in center-based child care.

**Table 2:** Prevalence of child care according to sample characteristics (n = 3,870).

Characteristic	child care attendance		
	Never n (%)	Some n (%)	Always n (%)
Sex			
Male	1,256 (65.1)	453 (23.5)	219 (11.4)
Female	1,275 (68.9)	400 (21.6)	175 (9.5)
Mother’s age			
≤ 20	542 (77.0)	130 (18.5)	32 (4.5)
21–30	1,244 (67.7)	402 (21.9)	192 (10.4)
≥ 31	745 (60.3)	321 (26.0)	170 (13.7)
Income			
1 (low)	559 (80.3)	110 (15.8)	27 (3.9)
2	559 (78.5)	116 (16.3)	37 (5.2)
3	477 (65.5)	169 (23.2)	82 (11.3)
4	461 (57.0)	222 (27.4)	126 (15.6)
5 (high)	321 (51.9)	192 (31.1)	105 (17.0)
Maternal education (years)			
0–4	289 (87.3)	34 (10.3)	8 (2.4)
5–8	786 (81.3)	147 (15.2)	34 (3.5)
9–11	898 (67.5)	306 (23.0)	127 (9.5)
≥ 12	557 (48.5)	366 (31.9)	225 (19.6)
Pre term (< 37 weeks)			
Yes	395 (70.8)	119 (21.3)	44 (7.9)
No	2,136 (66.3)	734 (22.8)	350 (10.8)
Low birth weight (< 2,500g)			
Yes	257 (71.6)	78 (21.8)	24 (6.7)
No	2,273 (66.5)	775 (22.7)	369 (10.8)
Maternal depression (3m)			
No	2,205 (66.1)	769 (23.1)	362 (10.8)
Yes	305 (74.6)	72 (17.6)	32 (7.8)
Storytelling (2y)			
No	1,393 (77.2)	290 (16.1)	121 (6.7)
Yes	1,138 (57.7)	563 (28.5)	273 (13.8)
Child visits parks (2y)			
No	1,186 (74.5)	286 (17.9)	121 (7.6)
Yes	1,345 (61.6)	567 (25.9)	273 (12.5)
Child visits other houses (2y)			
No	393 (68.5)	131 (22.8)	50 (8.7)
Yes	2,138 (66.7)	722 (22.5)	34 (10.8)
Participation in PIM (2y)			
No	2,227 (65.2)	804 (23.5)	386 (11.3)
Yes	304 (84.2)	49 (13.6)	8 (2.2)

Mean development score at two years was: cognitive (68.3; SD±14.5), language (61.4; SD±21.5), fine motor (90.0; SD±13.2), and gross motor (79.2 SD±13.4). Table 3 shows that girls had higher scores than boys for cognitive, language and fine motor development, and the opposite is true for gross motor skills, where boys presented higher values. Income and maternal education had a positive relation with cognitive and language outcomes – children of richer and more educated mothers presented higher scores of development. Maternal depression was negatively associated with language development. Children born preterm and with low birth weight presented lower means in all outcomes, except gross motor skills. Children whose parents read/told stories, took them to the park and to other peoples’s houses had higher scores for cognitive and language domains. Also, storytelling is positively associated with fine motor skills, while visits to other people’s houses were associated with both fine and gross motor skills. Participation in the PIM program was negatively associated with language development.

**Table 3:** Association between early childhood development at two years and sample characteristics (n = 3,870).

Characteristic	DEVELOPMENT			
	COGNITIVE β (95%CI)	FINE MOTOR β (95%CI)	GROSS MOTOR β (95%CI)	LANGUAGE β (95%CI)
Sex				
Male	0	0	0	0
Female	2.02 (1.10–2.93)	1.01 (0.18–1.84)	-1.72 (-2.56– -0.88)	6.86 (5.52–8.20)
Mother’s age				
≤ 20	0	0	0	0
21–30	0.92 (-0.33–2.17)	-0.61 (-1.74–0.52)	0.24 (-0.91–1.40)	-0.10 (-1.95–1.75)
≥ 31	0.98 (-0.34–2.32)	-1.41 (-2.61– -0.20)	0.16 (-1.06–1.39)	-0.66 (-2.63–1.31)
Income				
1 (low)	0	0	0	0
2	-1.05 (-2.53–0.44)	-0.50 (-1.87–0.86)	-0.40 (-1.78–0.98)	0.88 (-1.31–3.08)
3	1.45 (-0.02–2.93)	0.23 (-1.13–1.59)	1.43 (0.06–2.81)	3.03 (0.83–5.22)
4	2.55 (1.11–4.00)	0.57 (-0.76–1.90)	0.67 (-0.68–2.01)	4.63 (2.49–6.77)
5 (high)	5.14 (3.61–6.68)	1.16 (-0.25–2.58)	0.83 (-0.60–2.66)	8.43 (6.15–10.71)
Maternal education (years)				
0–4	0	0	0	0
5–8	0.41 (-1.36–2.18)	1.31 (-0.31–2.92)	0.14 (-1.51–1.79)	3.61 (0.99–6.23)
9–11	2.21 (0.50–3.92)	0.70 (-0.86–2.26)	0.81 (-0.78–2.40)	5.73 (3.20–8.26)
≥ 12	5.02 (3.29–6.76)	1.57 (-0.02–3.15)	0.55 (-1.07–2.16)	10.03 (7.46–12.59)
Pre term (< 37 weeks)				
No	0	0	0	0
Yes	-3.28 (-4.57– -1.99)	-3.02 (-4.18– -1.85)	0.27 (-0.92–1.46)	-6.07 (-7.97– -4.16)
Low birth weight (< 2,500g)				
No	0	0	0	0
Yes	-2.64 (-4.20– -1.09)	-2.03 (-3.44–-0.62)	-0.13 (-1.57–1.31)	-5.15 (-7.45– -2.44)
Maternal depression (3m)				
No	0	0	0	0
Yes	-1.44 (-2.91–0.04)	-0.65 (-1.99–0.69)	-0.29 (-1.65–1.07)	-3.62 (-5.80– -1.44)
Storytelling (2y)				
No	0	0	0	0
Yes	3.15 (2.24–4.07)	1.43 (0.60–2.26)	0.43 (-0.41–1.28)	5.80 (4.45–7.15)
Child visits parks (2y)				
No	0	0	0	0
Yes	1.97 (1.04–2.89)	-0.11 (-0.95–0.73)	0.44 (-0.42–1.29)	2.36 (0.99–3.74)
Child visits other houses (2y)				
No	0	0	0	0
Yes	3.19 (1.93–4.46)	1.18 (0.02–2.33)	1.87 (0.69–3.04)	4.35 (2.47–6.23)
Participation in PIM (2y)				
No	0	0	0	0
Yes	-1.05 (-2.61–0.51)	-0.82 (-2.23–0.60)	0.76 (-0.68–2.20)	-3.80 (-6.12– -1.49)

Table 4 presents crude and adjusted analyses of the association between child care attendance and child development. In crude analyses, attending child care centers was associated with higher cognitive and language development, with highest mean scores among children who always attended child care centers. In fully adjusted models (Model II including stimulation markers as well as other covariates), attending child care centers was still associated with higher cognitive development scores, again with the highest scores being most evident for children who always attended child care.

**Table 4:** Association between center-based child care attendance and early childhood development at 2 years.

	Crude		Model I		Model II	
	BETA	(95%CI)	BETA	(95%CI)	BETA	(95%CI)
Cognitive development		< 0.001*		< 0.001*		0.001*
Never	0		0		0	
Some point in life	2.53	(1.41–3.65)	1.67	(0.50–2.85)	1.35	(0.17–2.53)
Always	4.21	(2.68–5.74)	2.87	(1.26–4.78)	2.44	(0.83–4.05)
Fine motor skills		0.18*		0.25*		0.48*
Never	0		0		0	
Some point in life	0.60	(-0.42–1.61)	0.52	(-0.56–1.61)	0.32	(-0.78–1.41)
Always	0.72	(-0.66–2.11)	0.69	(-0.79–2.17)	0.44	(-1.05–1.93)
Gross motor skills		0.86*		0.68*		0.54*
Never	0		0		0	
Some point in life	-0.47	(-1.51–0.57)	-0.55	(-1.66–0.55)	-0.64	(-1.76–0.47)
Always	0.21	(-1.22–1.63)	0.01	(-1.51–1.52)	-0.13	(-1.65–1.39)
Language development		< 0.001*		0.04*		0.24*
Never	0		0		0	
Some point in life	2.16	(0.50–3.82)	0.53	(-1.19–2.24)	-0.17	(-1.90–1.55)
Always	5.26	(2.99–7.53)	2.68	(0.33–5.02)	1.83	(-0.52–4.19)

Crude analysis (n = 3,778); Model I: adjusted for sex, maternal age, family income, maternal education, gestational age, low birth weight, and maternal depression (n = 3,561); Model II: Model I + stimulation variables (n = 3,561); * Test for trend.

## DISCUSSION

This study showed that Brazilian children who attended child care centers between birth and two years of age had higher cognitive development scores. These associations remained even after adjustment for several confounders, including indicators of child stimulation, such as being read to or going to parks, which is an important determinant of early child development^17^.

Child development measured as cognitive and language scores presented similar patterns of associations with several other variables in the study (such as family income, maternal education, child sex, birth weight, gestational age and stimulation variables), but language was also associated with maternal depression and child participation in the PIM program. The negative association between low-birth weight, preterm birth, and all developmental outcomes, except for gross motor skills, is consistent with theories that prenatal programming and exposures may influence ECD and health^18^. Although maternal depression symptoms were not associated with other domains of development in the current study, language development was negatively associated with this condition. Other studies show that children born of depressed mothers show poorer socio-emotional and cognitive outcomes^19^, with maternal depression leading to compromised interaction patterns and reduced child stimulation, that may affect language development. The negative association found between PIM participation and language development in this study is likely to be explained by the fact that the program focuses on vulnerable families with children at elevated risk for developmental delay^20^.

In crude analysis, child care attendance was positively associated with cognitive and language development scores, but not motor development. Similar findigs have been reported in previous studies^6,8,9,21^. After adjustment for covariates, child care attendance was still associated with cognitive development, with the highest scores being found among children who always attended child care, rather than this being reported at only one point in time. Other studies have also shown long-term positive influence of child care attendance on cognitive outcomes^6,7^.

These results are similar to other studies^6,7,9,21^, which also found that children who attend any child care education program have better cognitive outcomes. Previous studies that investigated the impact of cash transfer programs and nutritional interventions found positive effects on cognitive outcomes. However, educational interventions such as center-based child care tend to have higher effect sizes for cognition than these other types^7^. Conversely, a systematic review about early childhood care and cognitive development has shown that the relationship between timing and duration of child care and cognitive development is still inconsistent^8^. Hence, our results suggest that children who have spent more time in center-based child care since birth have better cognitive scores, providing evidence that early entry in child care and its continuity may help improve children’s cognition.

Other studies have also shown that child care has positive effects on language development^9^, however, this effect may disappear after controlling for socio-economic status, home environment, and child stimulation^22^, which we observed in this study. Different activities related to stimulation, such as going to parks, or to other people’s houses, involving talking and interacting with other people, may be more related to language development than group-based child care and it may explain the lack of association in the final analyses of our study.

Several studies show that child stimulation – here measured in crude terms as storytelling, going to the parks or other people’s houses, and PIM participation – is a positive influence on child development^3,17^. This indicates that child care attendance may be an opportunity for stimulating development, even if the child is not stimulated at home.

Child care attendance was not associated with motor development. Other studies are mixed on this association – one did not find association^23^, and others reported positive associations^24,25^. It appears that only child care centers with interventions focused on movement activities, with adequate equipment and care, can improve motor development^25,26^.

In Brazil, one of the goals of the National Plan of Education (PNE – *Plano Nacional de Educação*) is expanding the provision of early childhood education centers to reach at least 50% of children aged up to three years^27^. In this study, about 1/3 of boys and girls attended child care. This proportion is low, considering the government’s goals, but is higher than the prevalence of child care attendance in the country as a whole (23.2%) and in the city of Pelotas (22.0%)^28^. Also, one study with children in state public child care centers in the country did not find any significant impact on cognitive or executive functions^10^, which may be due to the quality of care provided in the country, in which children who attended better care had greater benefits than those who did not^29^. Unfortunately, the type of child care (public or private and how much time per day children spent in the care) were not available for both follow-ups in our study, which did not allow its use alongside the exposure variable created for this study.

Despite the benefits of child care centers for children’s development, each child has his or her own individuality, to which center-based care may not respond to, as activities are organized for groups. Besides that, even though they may have appropriate training and preparation, the center caregivers do not have the same intimate relationship with the children as do parents or relatives and this can affect the capacity to interpret children signs and needs^30^. Moreover, parents, family, and home environments play a crucial role in ECD, and this always needs to be considered when interpreting child development, especially in situations of vulnerability^17^.

As with all observational studies, some limitations should be considered. There may have been some biases due to missing data. However, children with valid data did not differ substantially from the whole cohort. Also, we cannot discard residual confounding, since we did not measure other aspects important to ECD, related to the external child care and the home and family environment. The low magnitude of association (about 0.15 standard deviation of the cognitive development score), could be interpreted to mean low clinical relevance. However, in this critical period for human development, even small differences in neurodevelopment scores could signal the onset of long-term developmental processes, which reinforces the clinical relevance of our findings. Considering the strengths, this study used a valid measure of ECD, had a large sample size and used information from two follow-ups of the 2015 Pelotas Birth Cohort Study to build a longitudinal exposure variable. Also, the longitudinal design of the study allows improved temporal sequencing between the variables.

## CONCLUSIONS

Center-based child care attendance may help improve cognitive development, even after adjustment for diverse characteristics, including stimulation at home, and should be generally encouraged at this age. The effect was higher for children who had early and continued exposure. Considering that more than half of the sample never attended child care, it is important to advise parents about its relevance, and to invest in child care as a public policy to provide this opportunity for all.
